# A Data-Free Digital Platform to Reach Families With Young Children During the COVID-19 Pandemic: Online Survey Study

**DOI:** 10.2196/26571

**Published:** 2021-06-28

**Authors:** Linda Marleine Richter, Sara Naomi Naicker

**Affiliations:** 1 DSI-NRF Centre of Excellence in Human Development University of the Witwatersrand Johannesburg South Africa

**Keywords:** families, parenting, children, COVID-19, digital, survey, data-free

## Abstract

**Background:**

The COVID-19 pandemic and containment measures have severely affected families around the world. It is frequently assumed that digital technologies can supplement and perhaps even replace services for families. This is challenging in conditions of high device and data costs as well as poor internet provision and access, raising concerns about widening inequalities in availability of support and consequent effects on child and family outcomes. Very few studies have examined these issues, including in low- and middle-income countries.

**Objective:**

The study objectives were two-fold. The first objective was to gather data on the impact of the COVID-19 pandemic on families of young children using an online survey. The second objective was to assess the feasibility of using a data-free online platform to conduct regular surveys and, potentially, to provide support for parents and families of young children in South Africa.

**Methods:**

We used a data-free mobile messenger platform to conduct a short digital survey of the impact of the COVID-19 pandemic on caring for young children in South Africa. We report on the methodological processes and preliminary findings of the online survey.

**Results:**

More than 44,000 individuals accessed the survey link and 16,217 consented to the short survey within 96 hours of its launch. Respondents were predominantly from lower classes and lower-middle classes, representing the majority of the population, with urban residential locations roughly proportionate to national patterns and some underrepresentation of rural households. Mothers comprised 70.2% (11,178/15,912) of respondents and fathers comprised 29.8% (4734/15,912), representing 18,672 children 5 years of age and younger. Response rates per survey item ranged from 74.8% (11,907/15,912) at the start of the survey to 50.3% (8007/15,912) at completion. A total of 82.0% (12,729/15,912) of parents experienced at least one challenge during the pandemic, and 32.4% (2737/8441) did not receive help when needed from listed sources. Aggregate and individual findings in the form of bar graphs were made available to participants to view and download once they had completed the survey. Participants were also able to download contact details for support and referral services at no data cost.

**Conclusions:**

Data-free survey methodology breaks new ground and demonstrates potential not previously considered. Reach is greater than achieved through phone surveys and some social media platforms, men are not usually included in parent surveys, costs are lower than phone surveys, and the technology allows for immediate feedback to respondents. These factors suggest that zero-rated, or no-cost, services could provide a feasible, sustainable, and equitable basis for ongoing interactions with families of young children.

## Introduction

The COVID-19 pandemic has affected families everywhere, both directly through illness and death, and indirectly through the effects of containment measures on economic activities and routines of daily life. Lockdowns, with varying degrees of restriction, have been imposed in many countries, and by the end of March 2020, more than 20% of the world’s population was estimated to be under lockdown [1]. Many countries are, or will soon be, under second or third lockdowns. Although SARS-CoV-2 has had less serious effects on the morbidity and mortality of young children compared to older age groups, preschool children have been severely affected by indirect effects [2].

In South Africa, as in many other countries, early childhood development centers, public nurseries, kindergartens, and preschools have been closed since late March 2020 under one of the strictest lockdowns in the world. Private facilities began to reopen in late July 2020, but by August 2020, only 13% of children under the age of 6 years were attending their usual facility [3]. Movement restrictions and bans on visiting between households meant that families were not able to draw on the assistance of relatives and friends for relief childcare. As a consequence, families have had the sole responsibility for providing nurturing care for young children 24 hours a day, ensuring children’s good health and nutrition, safety and security, and early learning opportunities as well as providing love and affection [4].

Among exacerbating concerns about childcare, some 3 million South Africans are estimated to have lost their jobs as a result of the effects of the COVID-19 pandemic on the economy, with women most affected [5]. Inestimable numbers of informal workers, mostly women, also lost their ability to generate income. As only 34% of South African children live with both parents [6], mothers, grandmothers, and aunts carry a heavy burden for both childcare and financial support for young children. Providing educational input for older siblings under school closures is an added responsibility for many families, given that most households have more than one child [7]. Confinement in small, crowded living spaces, together with fear of infection, are adding to mental health stresses in South Africa as in other countries, with potentially further adverse effects on children [8], particularly younger children [9].

Both short- and long-term adverse physical, psychological, and social effects of the pandemic conditions on children are predicted, with supporting evidence emerging. These include interrupted, delayed, and missed preventive health care visits for pregnant women and children, separation of parents and neonates at birth, closure of day care facilities, household poverty and food shortages, parental and child mental health stresses, and increased risk of parental substance abuse and interpersonal violence, including child abuse [10]. Ongoing longitudinal studies confirm that parental mental health has deteriorated, that children are more irritable and sleep less [11], that younger children are more likely than older children to manifest symptoms such as clinginess and fear that family members might become infected [12], and that women and working parents are finding it hardest to cope [13].

There is considerable optimism about the potential application and expansion of digital technologies to fill gaps in knowledge and supplement health and social care during the pandemic [14]. These include public communication using mobile phones and the internet, surveys, digital surveillance and contact tracing, electronic clinical monitoring, telehealth, and counseling services [15]. Along these lines is a proliferation of digitally delivered parental guidance, advice, activities, and learning materials produced by governments, civil society groups, and multinational organizations to help maintain healthy adult-child interactions to support young children’s development [16,17]. Digital technologies and methods are also being used to investigate the effects of pandemic conditions on families and young children. These include surveys delivered by phone and video, Facebook, Instagram, and Twitter [18-20].

While important efforts are being made to understand the effects of the COVID-19 pandemic on parents and young children using digital technologies, a number of challenges have to be addressed. Among these are that few studies have specifically looked at effects on preschool children; survey samples tend to be small, undefined, and/or selective, and questionnaires tend to be long, taking 40 minutes to an hour to complete [19,21]. Further, one-off surveys close to the start of initial lockdowns likely underestimated the long-term effects on children through continued job losses in families, increased household poverty, chronic parental mental health problems, and repeat lockdowns. What would be most helpful are repeat, tailored surveys to monitor compounding impacts on families, how family coping strategies evolve, and the interventions that give greatest relief at different stages of the pandemic’s impact.

In low- and middle-income countries, the most immediate challenge is to establish communication channels to reach the greatest number of affected families in order to convey accurate information on how families can protect themselves and their children, solicit the changing needs of families, and respond effectively to their needs. In this respect, it has been recognized that even in high-income countries, few of the most marginalized groups are reached by digital technologies, and that it is essential to develop tools to address gaps in internet access to avoid a COVID-19–related increase in inequality due to the “digital divide” [15,22]. According to UNICEF (United Nations Children’s Emergency Fund) [23], distance learning has failed to make up for school attendance, with about one-third of children in the countries surveyed not reached at all. Even in countries where distance learning exists, only two-thirds of children are reached by television and one-quarter by online delivery.

It is estimated that internet usage worldwide varies from approximately 87% in Europe to approximately 34% in Africa, with the lowest access (23%) among African women [24]. The most common reasons for lack of internet use are the high cost of devices and data, and poor provision and access to data services. Like many other countries, South Africa is highly unequal. Internet penetration is estimated at around 62%, with most people having access through their mobile phones. About double the number of users live in urban as compared to rural areas [25]. Only about 10% of South Africans have a stable internet source in their homes [26]. WhatsApp—a data-driven platform—is the most frequently used social media app, followed by Facebook (87%), Instagram (61%), and Twitter (44%) [25]. WhatsApp has evolved into one of the primary methods of communication between individuals and between communities, governments, and nongovernmental organizations (NGOs).

Some online surveys have been conducted to ascertain understanding, practices, and impacts of the COVID-19 pandemic among South Africans, although none specifically have focused on parents of young children or on young children themselves [27-29]. In order to survey large numbers of parents of preschool children to ascertain their most pressing needs during COVID-19 lockdown conditions and how families were coping, we trialed the use of a data-free, zero-cost social media platform. If successful, the platform and similar other channels could be used to establish ongoing communication with parents of young children in order to communicate prevention measures, survey COVID-19 impacts, and provide appropriately targeted interventions.

## Methods

### Study Design

We designed a short questionnaire consisting of between 18 and 30 questions, depending on response options, with one item displayed on-screen at a time and a progress indicator. Skip patterns and branching logic were used to streamline questions and improve participant experience by reducing the number of irrelevant questions requiring a response. The small number of questions also eliminated the need for their randomization. Participants were required to provide a response for each question to move forward in the survey and nonresponse options in the form of *other* were included, but participants could move backward to edit prior responses. The questionnaire was translated into the most common languages used in South Africa: Afrikaans, English, Sesotho, isiZulu, and Sepedi. The questionnaire and translated versions were programmed into REDCap (Research Electronic Data Capture), a secure web platform designed to support survey distribution and data capture for research [30,31]. A list of national referral and support services for families was uploaded in Adobe Acrobat format and made available to download at the conclusion of each completed survey.

A set of screening questions excluded participants younger than 18 years of age, those not caring for a child under 5 years of age, and those not living in South Africa. All participants were required to consent to the survey, as mandated by the Human Research Ethics Committee of the University of the Witwatersrand (H20/06/38). The informed consent process included disclosures of the nature and purpose of the survey, risks and benefits of participation, uses to which the data would be put, guarantees of anonymity, and investigator contact details as well as those of the responsible ethics committee. Demographic details were kept to a minimum to make the survey as short as possible and encourage participation. Questions covered challenges of caring for young children, sources and types of help received, as well as unmet needs. The questionnaire was piloted among staff and colleagues speaking each of the languages. The English version of the questionnaire is attached as Multimedia Appendix 1. Coding of multilingual responses was held constant to allow for integrated analysis and immediate graphic presentation of results.

We used the Moya Messenger platform, hosted by biNu (now called Datafree), as our population source for convenience sampling [32]. biNu’s technology offers two services: the first is to reverse-bill online content through partnerships with all major cellular networks in South Africa, and the second is the data-free Moya Messenger platform. Their Moya Messenger app is a growing platform of users who are able to send messages to other users without incurring data costs. The app offers unlimited texting, group chat, end-to-end security with automatic encryption, and contact discovery, similar to WhatsApp and Viber, but without the use of the individual’s data. The platform is monetized through a Moya Discover service where external parties pay to have their websites, surveys, and content featured. Surveys are pinned to the platform and open to all users subscribed to Moya. Users of the Moya platform are made aware of the terms and conditions associated with using the app, including exposure to advertising.

The data-free services are used in two ways. The first is to have all survey content reverse-billed. This generates a data-free link that can be shared through any medium so that participants can access and complete the survey without paying for data. The second is to share survey links with the sample of Moya subscribers who have access to the interface where the survey is pinned and are able to complete the survey without incurring any data costs. A grant awarded to the University of the Witwatersrand was billed for data used by participants at the rate of 20 South African cents (US $0.015) per megabyte, averaging R6 (US $0.44) per survey response. By February 2020, the Moya platform had in excess of 2.3 million active daily users [33] of all genders, age categories, and income groups. The user profile is 53% female and 90% so-called non-White, with 80% of the sample falling into a Living Standards Measure of between 3 and 7 deciles based on urbanization and asset ownership [34], and 92% earning less than R15,000 per month (US $1000). That is, users fall into lower-class and lower-middle-class groups.

### Distribution of the Survey

**Figure 1:**
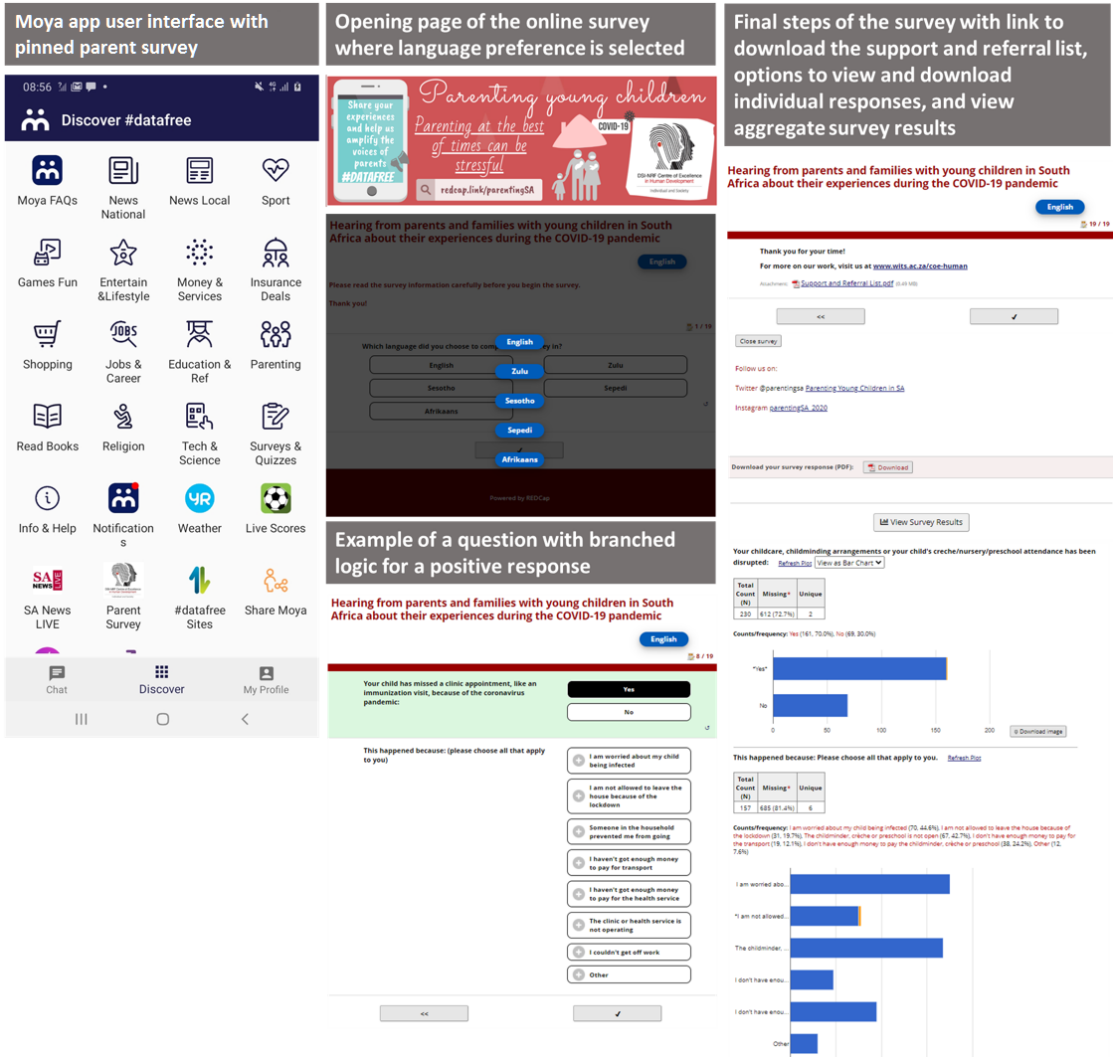
Screenshots of the online survey, including language selection, questions, and individual and aggregate findings, as pinned on the Moya app.

REDCap generated a URL link to the survey that could be distributed from the web platform or from other sources. biNu reconfigured the URL and all its content to be reverse-billed to a secure account held by the research team. All responses linked to the URL were transmitted directly to the REDCap server and collated in a secure database. For distribution on the Moya Messaging platform, biNu placed a pinned notice of the survey on the platform’s interface where users are able to view news, updates, and survey alerts. Since the survey link was available to any user of the platform, it was considered an open survey. The link was the initial contact with potential participants who were able to see the pinned notice on their user interface and could open it to complete it (Figure 1). Once the survey link was opened from the Moya interface, users were asked to choose their language preference and were directed to the information section of the survey, which detailed the purpose of the survey, eligibility, and consent and that there would be no incentive for participation. Each time the link was opened, a record of that response was created in REDCap as a single observation. The survey was not restricted to a single response per device. The survey was pinned to an app—Moya Messenger—and was only accessible from a device with the app installed. The absence of an incentive was also thought to discourage multiple entries from the same individual. Cookies were not collected, but REDCap did collect Internet Protocol addresses along with a master log file of all survey activity, which could be analyzed retrospectively to identify duplicate responses; however, this data are not accessible to normal end users and strict processes to ensure anonymity must be adhered to before REDCap grants access to this data. During the piloting phase, the survey took between 4 and 8 minutes to complete, with an average of 5 minutes. Accurate survey lengths could not be calculated during the data collection phase because participants were allowed to leave the survey and return at a later time to complete it.

### Analysis

Responses to the survey were collected and stored in REDCap in real time, allowing continuous online analysis of data. Once the responses reached time and budget allocations for the study, the survey was terminated on both the REDCap and Moya platforms. Data cleaning and quality control were undertaken on the REDCap platform using built-in data validation features. The data were exported into SPSS, version 26.0 (IBM Corp), for further data cleaning and analysis. Cases with missing data were not excluded from the analysis and no statistical corrections were performed to adjust for any nonrepresentativeness.

## Results

The progression of participation in the survey was tracked by calculating attrition at each stage of the screening questions. The survey link was opened by 44,292 users within 96 hours of it appearing on the data-free platform; 21,993 participants were of eligible age, 17,325 participants were caring for a child under the age of 5 years, and 16,217 participants consented to participate in the survey, at a recruitment rate of 36.6%. A total of 1.9% of respondents (305/16,217) were caregivers of groups of children in day care centers. These responses were excluded from the analysis, and 15,912 eligible consenting participants comprised the analytical sample.

Response rates and missing values are shown in Table 1. More than half of the participants (8007/15,912, 50.3%) responded to all questions.

**Table 1:** Response rates to survey questions and missing values.

Variable	Participants who responded (N=15,912), n (%)	Participants who did not respond (N=15,912), n (%)
Missed clinic visits	11,907 (74.8)	4005 (25.2)
Disruption in ECD^a^ services	10,646 (66.9)	5266 (33.1)
Breastfeeding challenges for children 0 to 6 months of age	1545 (73.8)	548 (26.2)^b^
Child-feeding challenges	9954 (62.6)	5958 (37.4)
Difficult to be affectionate	9310 (58.5)	6602 (41.5)
Violence toward child	9001 (6.6)	6911 (43.4)
Child behavior challenges	8668 (54.6)	7244 (45.5)
Receiving community help	8439 (53.0)	7473 (46.9)
Receiving government help	8246 (51.8)	7666 (48.2)
Receiving nongovernmental organization help	8007 (50.3)	7905 (49.7)

^a^ECD: early childhood development.

^b^Calculated from a total of 2093 children aged 0 to 6 months of age.

By residence, respondents were roughly representative of the South African urban population residing in cities, suburbs, and townships, with lower representation from rural respondents who have less access to mobile phones and the internet (Table 2 [35,36]). By 2016, 99% of South Africans in urban areas had a smartphone, compared to 83% in rural areas [37]; however, only 45% of rural households were able to access the internet using their mobile devices, compared to 64% of urban households [38].

**Table 2:** Representativeness of the sample by area of residence.

Area of residence	Participants (n=15,204), n (%)	National average, %
City or suburb	4959 (32.6)	27
Township^a^	6578 (43.3)	40 [35]
Rural settlement, village, farm, or tribal area	3068 (20.2)	33 [36]
Other	599 (3.9)	N/A^b^

^a^Townships were created as segregated dormitory suburbs in urban areas to house African workers under Apartheid.

^b^N/A: not applicable; an *other* category was added to this survey but is not included in the national census. A nonspecific response option is generally recommended, especially if respondents are required to give a response before moving on to the next question.

Although the majority of South Africans are African-language speakers, 83.0% of respondents (13,207/15,912) completed the survey in English, the main language of instruction in South African schools, following the teaching of mother tongues in Grades 1 to 3. A substantial proportion of participants were fathers (4734/15,912, 29.8%).

Table 3 shows the age and gender of children to whom respondents referred in the survey. Children were roughly evenly divided between those aged 0 to 3 years and those aged older than 3 to 5 years, as well as between boys and girls. A total of 18,672 children were included in the analysis, but questions were not answered in reference to a single index child. About half of the parents (6799/13,228, 51.4%) reported 1 child under 5 years of age in the home, and 40.9% (5125/12,522) reported 2 to 3 children under 5 years of age in the home.

**Table 3:** Characteristics of children to whom respondents referred in the survey.

Characteristic			Children, n (%)	Cumulative %		
**Age (n=18,238)**						
	0 to 6 months	2093 (11.5)			11.5	
	>6 months to <1 year	2252 (12.4)			23.8	
	1 to 3 years	4954 (27.2)			50.9	
	>3 years to 5 years	8939 (49.0)			100	
**Gender (n=18,672)**						
	Female	9361 (50.1)			50.1	
	Male	9311 (49.9)			100	

Responses to the survey questions (Table 4) showed that families were severely affected by the government’s attempts to contain the COVID-19 pandemic. The detailed results of the effects of COVID-19 on families are under preparation. One-third of children (3920/11,907, 32.9%) were reported to have missed an immunization visit, mainly because parents feared that their child would become infected; 68.7% (7313/10,646) of children’s day care and crèche arrangements were discontinued or disrupted, also mainly because of fear of infection. This response was fairly constant from parents with children 0 to 6 months of age to 3 to 5 years of age, indicating that closure of services and facilities also affected parents with very young infants. One-third of mothers (367/1049, 35.0%) and fathers (117/403, 29.0%) reported that breastfeeding a child under 6 months of age was difficult, citing fear of infecting their baby. Half of all parents (4964/9954, 49.9%) were finding it difficult to feed their young child, mainly because the family did not have enough money to buy appropriate food. A total of 41.2% of parents (3832/9310)—proportionately more fathers (1150/2419, 47.5%) than mothers (2372/6135, 38.7%)—were finding it difficult to be affectionate toward their child, due to an even division between parental stress and depression, household tension, and child irritability and crying. Close to one-third of parents (1662/5760, 28.9%), slightly more mothers, were finding it difficult to deal with their young child’s behavior. Responses as to how parents were coping ranged from trying to comfort a distressed and crying child (960/2391, 40.2%), punishing a child for being naughty (745/2391, 31.2%), feeling hopeless and not knowing what to do (764/2391, 31.9%), and asking other household members for help to distract and comfort a child (239/2391, 10.0%). A total of 13.8% of fathers (320/2314) and 11.0% of mothers (657/5961) reported that someone in the household had been angry and violent toward their child. Most often, violence was reported to be perpetrated by another adult in the household (720/1123, 64.1%), but 17.7% (60/339) of fathers and 12.4% (85/687) of mothers reported that they had been angry and violent toward their child. The most frequent reasons given for getting angry and violent with a young child was when an adult lost their temper (469/1070, 43.8%), when the child broke or took something they were not supposed to touch (350/1070, 32.7%), and to prevent the child from being hurt or injured by, for example, fire, poison, or an open water source (173/1070, 16.2%).

Two-thirds of parents said they needed help, the majority of whom were in urgent need of money, vouchers, or food parcels. Clothes; blankets; personal protective equipment (PPE), such as masks and soap; and medicine were also high on their list of needs. When asked what help they had received from different sources, more parents reported getting assistance from governmental organizations (2581/8246, 31.3%) than from neighbors and community groups (2028/8439, 24.0%) or from NGOs (1106/8007, 13.8%). Among those who did receive help, the most common form was reported to be money or vouchers from governmental organizations (1175/3349, 35.1%). Help received from NGOs was most frequently reported to be food parcels (612/1619, 38.8%). Neighbors and community groups were reported to give a wide range of help, most commonly food, financial loans, PPE, information, relief childcare, and emotional support for mental distress.

**Table 4:** Responses regarding challenges and support by parent type.

Survey item			Mothers, n (%)		Fathers, n (%)	Total, n (%)^a^
**I have missed a clinic appointment, like an immunization visit, because of the coronavirus pandemic**						
	Total	7468 (100)		3422 (100)		11,907 (100)
	Yes	2446 (32.8)		1165 (34.0)		3920 (32.9)
	No	5022 (67.2)		2257 (66.0)		7987 (67.1)
**My childcare, childminding arrangements, or my child's crèche, nursery, or preschool attendance has been disrupted**						
	Total	6845 (100)		2911 (100)		10,646 (100)
	Yes	4641 (67.8)		2027 (69.6)		7313 (68.7)
	No	2204 (32.2)		884 (30.4)		3333 (31.3)
**Breastfeeding my baby is difficult during this time**						
	Total	1049 (100)		403 (100)		1545 (100)
	Yes	367 (35.0)		117 (29.0)		510 (33.0)
	No	682 (65.0)		286 (71.0)		1035 (67.0)
**I am struggling to properly feed my young child**						
	Total	6489 (100)		2636 (100)		9954 (100)
	Yes	3185 (49.1)		1407 (53.4)		4964 (49.9)
	No	3304 (50.9)		1229 (46.6)		4990 (50.1)
**It is difficult to be affectionate with my child during this time**						
	Total	6135 (100)		2419 (100)		9310 (100)
	Yes	2372 (38.7)		1150 (47.5)		3832 (41.2)
	No	3763 (61.3)		1269 (52.5)		5478 (58.8)
**Someone in my household has been angry and violent toward my child**						
	Total	5961 (100)		2314 (100)		9001 (100)
	Yes	657 (11.0)		320 (13.8)		1070 (11.9)
	No	5304 (89.0)		1994 (86.2)		7931 (88.1)
**The angry and violent person was:**						
	Total	687 (100)		339 (100)		1123 (100)
	You	85 (12.4)		60 (17.7)		157 (14.0)
	Another adult	454 (66.1)		205 (60.5)		720 (64.1)
	Another child	148 (21.5)		74 (21.8)		246 (21.9)
**I find my child more difficult to deal with**						
	Total	5760 (100)		2209 (100)		8668 (100)
	Yes	1662 (28.9)		532 (24.1)		2391 (27.6)
	No	4098 (71.1)		1677 (75.9)		6277 (72.4)
**I have received help from my neighbors, community, or faith groups**						
	Total	5625 (100)		2137 (100)		8439 (100)
	Yes	1332 (23.7)		532 (24.9)		2028 (24.0)
	No	2759 (49.0)		1032 (48.3)		4085 (48.4)
	I don’t need help	1534 (27.3)		573 (26.8)		2326 (27.6)
**I have received help from the government**						
	Total	5500 (100)		2082 (100)		8246 (100)
	Yes	1849 (33.6)		536 (25.7)		2581 (31.3)
	No	3137 (57.0)		1359 (65.3)		4867 (59.0)
	I don’t need help	514 (9.4)		187 (8.9)		798 (9.7)
**I have received help from nongovernmental organizations**						
	Total	5341 (100)		2021 (100)		8007 (100)
	Yes	687 (12.9)		319 (15.8)		1106 (13.8)
	No	4135 (77.4)		1508 (74.6)		6097 (76.2)
	I don’t need help	519 (9.7)		194 (9.6)		804 (10.0)
**What kind of help do you need most to look after yourself and your child?**						
	Total	13,788 (100)		5534 (100)		19,232 (100)
	Food parcels	2893 (21.6)		1035 (18.7)		3928 (20.4)
	Clothes and blankets	1588 (11.5)		548 (9.9)		2136 (11.1)
	Medicine	1180 (8.6)		527 (9.5)		1707 (8.9)
	Information	504 (3.7)		288 (5.2)		792 (4.1)
	Masks, soap, sanitizer, and gloves to protect us from the coronavirus	1533 (11.1)		616 (11.1)		2149 (11.2)
	Clean water	540 (3.9)		290 (5.2)		830 (4.3)
	Money or vouchers	3016 (21.9)		1258 (22.7)		4274 (22.2)
	Childcare	595 (4.3)		244 (4.4)		839 (4.4)
	Help in the home	327 (2.4)		131 (2.4)		458 (2.4)
	Transport to the clinic or to the shop	314 (2.3)		158 (2.9)		472 (2.4)
	Protection from someone in the house who is violent	162 (1.2)		91 (1.6)		253 (1.3)
	Support for mental distress, such as counseling	628 (4.5)		216 (3.9)		844 (4.4)
	I don’t need any help	418 (3.0)		132 (2.4)		550 (2.9)

^a^Total values exceed the sum of values for mothers and fathers since they include responses that have not specified parent type.

## Discussion

### Principal Findings

We assessed the feasibility of an online survey delivered through a data-free platform to investigate the variability of challenges facing families of young children. We restricted the survey to individuals living in South Africa and to adults caring for children 5 years of age and younger at home. In this paper, we report on the strengths and weaknesses of the use of an online survey in general, and of a data-free platform in particular, to monitor COVID-19 effects on families over time. This is an important question, given the likely long-term aftereffects of the pandemic on daily life [28] and the generally high cost of devices and data and, consequently, low rate of internet access in sub-Saharan African. South Africa will remain under varied levels of lockdown throughout 2021, which includes an overnight curfew, mandatory mask wearing, social distancing, and restrictions on gatherings. The government has issued directions of “conditions of return” for day care centers and preschools, including screening, masks for children over 2 years of age, clearly indicated spacing between children, and smaller staff to child ratios [39]. Financial losses and a likely very slow economic recovery mean that the shocks of the pandemic will be felt for the greater part of early and middle childhood for this cohort of children. Families who continue to get poorer may be forced to leave their homes to live with relatives; remove children from preschool and school because they cannot afford fees, transport, and supplies; and send one or more children to live with family in other parts of the country, as has occurred during other crises endured on the subcontinent. This study was able to rapidly and cost-effectively gather data from a large sample on a relatively broad range of challenges affecting families with young children with no cost to participants. One-off surveys administered in the early stages of lockdown must be repeated over time to track cumulative effects on children over the next 4 to 5 years. The University of Oregon’s RAPID-EC (Rapid Assessment of Pandemic Impact on Development–Early Childhood) study [40] and the University of Oxford’s Co-SPACE (COVID-19 Supporting Parents, Adolescents, and Children in Epidemics) study [41] are two examples of ongoing, large-scale, repeat online surveys including parents and young children.

We were not able to locate any published COVID-19 surveys that focused on young children and that were delivered on a data-free online platform, a gap that this paper attempts to fill. Many surveys used phone or email interviews [42] or social media platforms, such as Facebook, Twitter, and LinkedIn [18,43], to deliver survey links. These methods are subject to a number of selective factors. They all presume existing paid internet use and, in the case of phone interviews, a pool of what are often frequently changing mobile telephone numbers [44].

In contrast, online data-free surveys cast a wide net and arguably reach those most affected by the pandemic, as demonstrated through the findings of our study. This reach is dependent on the availability of telecommunication entities with the capacity to partner with multiple major networks in a given country to offer reverse-billing services. The additional user pool that the Moya Messenger platform presented—users who were familiar with the survey alert system—contributed to the high response rate. Efforts to share the survey links on Twitter and other social media sites garnered drastically fewer responses without a strong and sustained communication strategy and networks with access to large groups. Eligibility criteria led to a large drop-off of respondents, suggesting that users of zero-rated (ie, no-cost) services “cruise” around looking for topics of interest to them and, most likely, those that offer incentives. The effects of incentives on response rates and data quality have long been debated, particularly in the context of online surveys where control of multiple responses is much more complicated. The offset costs of online surveys, compared to traditional data collection methods, may encourage the use of incentives for respondents, particularly with some evidence that incentives increase response rates without reducing data quality [45].

In contrast to telephone interviews, data-free online surveys are very cheap. We received 15,912 surveys at a cost of R110,000 (US $7333), including setup costs, or R6.9 (US $0.46) per survey. By our calculation, using current rates for interviewers, training, and telephone supervision costs, as well as second or third attempts, approximately 20% of the time, to get an answer from calling telephone numbers [44], a single 20-minute phone interview in South Africa would cost around R80 (US $5.33). Repeat surveys using online data-free surveys are, therefore, feasible and affordable. A sample such as the one available on the Moya user platform offers a ready group of potential respondents who are familiar with surveys. While there are disadvantages to the use of a single, albeit large, convenience sample such as this, there are also advantages. The closed nature of the sample allows for easier penetration for repeat surveys, the sample is well-defined based on user demographics analyzed by the host entity, and, specifically for Moya, the data-free service attracts those in groups who are most in need. Such a platform is valuable where the purpose is to rapidly and efficiently reach a large sample that can be generalized to a larger proportion of the population and to collect data that can be quickly acted on to guide policy and practice, particularly in emergencies. Online surveys outside of such a platform and in the public sphere are equally useful, if not more so in terms of generalizability, but require substantially more time and effort to recruit potentially representative participants through social media platforms, television, radio and newspaper adverts, and databases of individuals. Accessing large databases of individuals raises ethical questions when beneficiaries and customers have not agreed to be solicited for participation in surveys, regardless of personal or societal benefits. Legal frameworks, including South Africa’s Protection of Personal Information law (Act 4 of 2013), are perhaps further along than ethical bodies governing digital research.

The use of online surveys offers a larger degree of anonymity compared to other data collection methods and may be more effective at eliminating social desirability bias for sensitive issues and at encouraging participation from those who would otherwise be reluctant. The wide reach of data-free online platforms is illustrated by the comparatively large number of fathers (30% of all respondents) who completed a survey about young children. It is notoriously challenging to engage male caregivers in parenting issues [46], and men are less likely to be targeted by phone surveys about family issues. Men’s perspectives on family challenges are important, as they are frequently the financial providers and decision makers.

**Figure 2:**
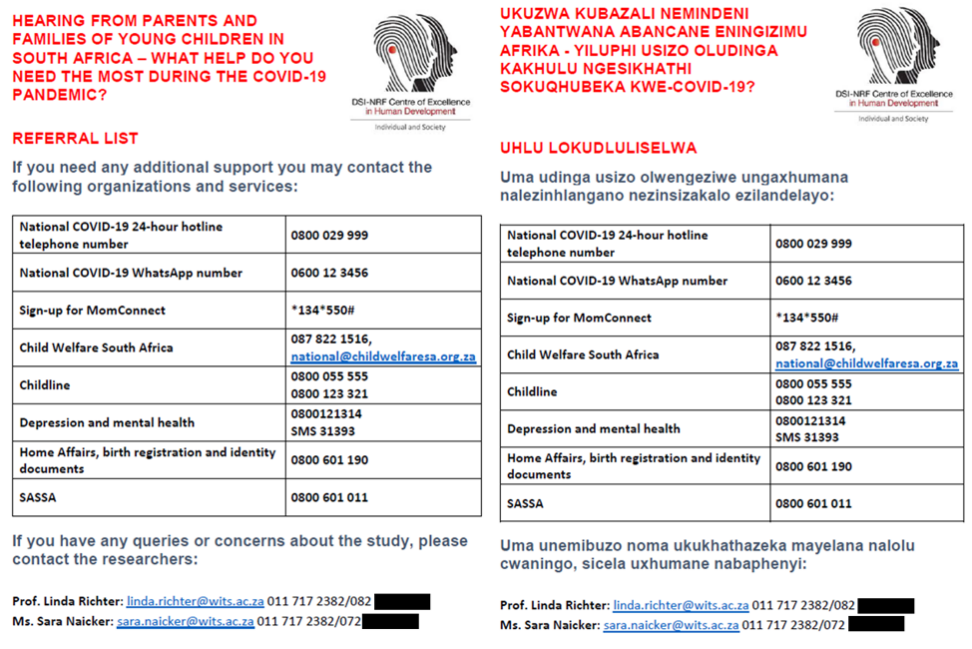
Support and referral services in English and isiZulu.

We were able to provide immediate feedback on response trends on the data-free platform for those respondents who were interested to look at them. This was made possible because the questionnaire was designed in REDCap [47] and survey responses were directed seamlessly back into the secure REDCap server, which provided individual and aggregate analyses of available data. In addition, a list of referral services for families needing immediate help was available for download at nil data costs once the survey was completed (Figure 2). We were also able to program the survey in additional languages, another feature of REDCap. In the South African context, where the poorest and hardest-to-reach groups are often those not fully literate in English, the additional, minor cost of translation warranted the effort for the 17% of the sample who chose to answer the survey in an African home language.

Digital and other technologies are advancing quickly to fill gaps created in information collection and service provision occasioned by the COVID-19 pandemic [48]. However, the danger of growing inequities due to differential access to the internet is acknowledged, an issue that is particularly pertinent in low- and middle-income countries. Data-free platforms supported by governments, external funders, and the private sector have the potential to expand internet access and can be used to monitor the effects of the pandemic, adapt supports, and create and expand two-way communications between families with young children and service providers. Data-free content that increases access to learning and knowledge has seen some growth during the pandemic, with universities and other institutions either subsidizing data costs or offering zero-rated websites. In the public domain, UNICEF’s Internet of Good Things [49] hosts mobile-packaged content designed to make content on many issues, from maternal health and positive parenting to sexual and reproductive health, available for free, even on low-end devices.

### Challenges and Limitations

As anticipated, families from rural areas were underrepresented compared to national population distributions. Rural households in South Africa and other countries have less access to smartphones, and even when these devices are present, access to the internet due to high data costs and available signal is lower than in urban areas. Data-free technology goes a long way to reaching rural families, but additional measures, such as WhatsApp and push message services, may be needed to close the gap further. Zero-rated services are not new. Concerns that they are not *net neutral* and that service providers can exercise control over content [50] need to be addressed to increase global internet access at a time when the COVID-19 pandemic has made it most needed.

We refrained from asking for detailed personal information (age, education, employment, race, etc) for fear of deterring respondents from completing the survey in the face of a long run-in of questions perceived to be less salient to the topic to which respondents were attracted [51]; that is, the challenges of caring for young children during the COVID-19 pandemic. Nonetheless, such information would be useful for more fine-grained analysis of the data. Further, our survey was designed as a single cross-sectional enquiry. Repeat surveys are critical in the context of the anticipated long-run consequences of the COVID-19 pandemic on families, and we did not test the willingness of respondents to be identified or to be anonymously resurveyed at a later time. Although we made the real-time results of the survey available on the platform, together with a list of referrals, we did not, at this time, monitor how many respondents accessed the results or downloaded the referral sources.

Many of the features of the online data-free survey described here are specific to the technology used by the researchers. Capabilities for programming multilingual surveys and revealing individual and aggregate findings instantaneously, among others, are not standard across the growing number of online survey platforms. In addition, the use of any individual feature is rarely without disadvantages on the flip side. For example, the option to prevent a single device from submitting multiple responses may prevent an individual from submitting multiple survey responses, but does not allow more than one eligible household member to complete the survey when relevant. Forced response options, which conventionally were thought to improve completeness of data, result in an individual dropping out of the survey altogether rather than missing individual items along the survey path. Researchers need to carefully consider the packages, platforms, and survey options against their research aims and objectives to ensure that the benefits of online surveys are fully realized and that disadvantages are minimized.

### Conclusions

Although digital technologies show tremendous promise to bridge gaps created by the suspension of face-to-face surveys and services, we have yet to come to grips with the very stark inequalities of internet access, both between and within countries. In this study, we demonstrate the feasibility and value of using a zero-rated service provider to conduct a survey of COVID-19 pandemic impacts on families of young children in a lower-middle-income country. The response rate was higher than comparable surveys, the survey was affordable, and it drew in a wide audience, demonstrated by the large number of fathers who participated. Further developments in digital services to respond to COVID-19 pandemic impacts, whether through surveys or online services such as counseling and education, need to consider using data-free platforms to ensure that the most vulnerable families are reached and can participate, and new sources of funding need to be opened up to do so.

## Appendix

Multimedia Appendix 1 English version of the South African parent survey.

## Abbreviations

Co-SPACE: COVID-19 Supporting Parents, Adolescents, and Children in Epidemics
NGO: nongovernmental organization
PPE: personal protective equipment
RAPID-EC: Rapid Assessment of Pandemic Impact on Development–Early Childhood
REDCap: Research Electronic Data Capture
UNICEF: United Nations Children’s Emergency Fund
