# Supplementary material for: A Data-Free Digital Platform to Reach Families With Young Children During the COVID-19 Pandemic: Online Survey Study
Source: JMIR Pediatr Parent. 2021 Jun 28;4(2):e26571. doi: 10.2196/26571 (PMC8244727; doi:10.2196/26571)
Supplement: Multimedia Appendix 1 [file pediatrics_v4i2e26571_app1.pdf]

Hearing from parents and families in South Africa about how to protect the youngest children during the COVID-19 pandemic

1. **We want to make sure that you have read and understand the information that we have given you about the survey:**
   ☐Yes, I understand
   ☐No, I don't understand

2. **You are 18 years or older:**
   ☐Yes
   ☐No

3. **You are the parent or caregiver of a child younger than 5 years:**
   ☐Yes
   ☐No

4. **You consent to participate in this survey:**
   ☐Yes
   ☐No

5. **The area you live in is best described as:**
   ☐City or town
   ☐Suburb
   ☐Township
   ☐Informal settlement in an urban area
   ☐Informal settlement in a rural area
   ☐Village or farm
   ☐Tribal area
   ☐Other ______________________________

6. **You are a:**
   ☐Mother
   ☐Father

7. **To how many children:** ________

8. **Please add the age and sex of each child:**
   ☐ 0-6 months' old ☐ boy ☐ girl
   ☐ 6 months - 1-year-old ☐ boy ☐ girl
   ☐ 1 year - 3 years' old ☐ boy ☐ girl
   ☐ 3 years - 5 years' old ☐ boy ☐ girl

9. **You have missed a clinic appointment, like an immunization visit, because of the coronavirus pandemic:**
 Yes
 No

10. **This happened because: (PLEASE CHOOSE EACH ONE THAT APPLIES TO YOU)**
 I am worried about my child being infected
 I am not allowed to leave the house because of the lockdown
 Someone in the household prevented me from going
 I haven’t got enough money to pay for transport
 I haven’t got enough money to pay for the health service
 The clinic or health service is not operating
 I couldn’t get off work
 Other ______________________________

11. **Your childcare, childminding arrangements or your child's crèche/nursery/preschool attendance been disrupted:**
 Yes
 No

12. **This happened because: (PLEASE CHOOSE EACH ONE THAT APPLIES TO YOU)**
 I am worried about my child being infected
 I am not allowed to leave the house because of the lockdown
 The childminder, crèche or preschool is not open
 I haven’t got enough money to pay for the transport
 I haven’t got enough money to pay the childminder, crèche or preschool
 Other ______________________________

13. **Breastfeeding my baby is difficult during this time:**
 Yes
 No

14. **This happens because (PLEASE CHOOSE EACH ONE THAT APPLIES TO YOU)**
 I am worried about infecting my baby
 People in my family have advised me not to give breastmilk to the baby
 My doctor or health provider advised me not to give breastmilk to the baby
 My milk is not enough
 I am stressed and my milk is not enough
 I don't have soap and clean water to clean my breasts
 Other ______________________________

15. **I am struggling to properly feed my young child:**
 Yes
 No

16. This happens because: (PLEASE CHOOSE EACH ONE THAT APPLIES TO YOU)

- There's not enough good food in the house because we don't have enough money
- There's not enough food in the house because I am scared to go out
- There's no transport to get food
- I don't have enough money for transport to get food
- No food is being sold or is available near where I live
- My child is unwell, restless or irritable and doesn't want to eat
- Other ______________________________

17. It is difficult to be affectionate to my child during this time:

- Yes
- No

18. This happens because: (PLEASE CHOOSE EACH ONE THAT APPLIES TO YOU)

- I am stressed and irritable
- I feel hopeless, I am depressed and withdrawn
- My child is crying a lot and is difficult to comfort
- There is a lot of tension in our house
- Other ______________________________

19. Someone in the household has been angry and violent towards my child:

- Yes
- No

20. This happened because: (PLEASE CHOOSE EACH ONE THAT APPLIES TO YOU)

- They lost their temper and hit my child
- My child was getting close to danger (fire, water, poison, etc.)
- My child broke or took something they weren't supposed to touch
- Other ______________________________

21. The angry and violent person was: (PLEASE CHOOSE EACH ONE THAT APPLIES TO YOU)

- You
- Another adult
- Another child

22. Do you find your child more difficult to deal with?

- Yes
- No

23. I am dealing with my child's behavior by: (PLEASE CHOOSE EACH ONE THAT APPLIES TO YOU)

- Punishing them because my child is being naughty
- Trying to comfort and calm my child
- Trying to get other people in the house to calm and distract my child
- I don't know what to do
- Other ______________________________

24. I have received help from my neighbours, community or faith group:

☐Yes
☐No
☐I don’t need help

25. What kind of help: (PLEASE CHOOSE EACH ONE THAT APPLIES TO YOU)

☐Food
☐Medicine
☐Information
☐Masks, soap, sanitizer, gloves to protect us from coronavirus
☐Lent or given me money
☐Helped with work around the house or yard, like cleaning, fetching water and so on
☐Transport to the clinic or shops
☐Looked after my child
☐Given me support for mental distress, e.g. sat with me, prayed for me
☐Other ______________________________

26. I have received help from government:

☐Yes
☐No
☐I don’t need help

27. What kind of help: (PLEASE CHOOSE EACH ONE THAT APPLIES TO YOU)

☐Food parcels
☐Clothes, blankets
☐Information
☐Medicine
☐Masks, soap, sanitizer, gloves to gloves to protect us from coronavirus
☐Given me clean water
☐Money or vouchers
☐Transport to the clinic or to the shop
☐Support for my mental distress, like counselling
☐Other ______________________________

28. I have received help from any non-government organizations:

☐Yes
☐No
☐I don’t need help

29. What kind of help: (TICK EACH ONE THAT APPLIES TO YOU)

☐Food parcels
☐Clothes, blankets
☐Information
☐Medicine
☐Masks, soap, sanitizer, gloves to protect us from coronavirus

- Given me clean water
- Money or vouchers
- Transport to the clinic or to the shop
- Support for my mental distress, like counselling
- Other ______________________________

30. What help do you most need to look after yourself and your child: (PLEASE CHOOSE EACH ONE THAT APPLIES TO YOU)

- Food
- Clothes, blankets
- Medicine
- Information
- Masks, soap, sanitizer, gloves to protect you from coronavirus
- Clean water
- Money or vouchers
- Child care
- Help in the home
- Transport to the clinic or to the shop
- Protection from someone in the house who is violent
- Support for your mental distress, like counselling
- Other ______________________________
- I don’t need any help

DISQUALIFICATION PAGE

UNDER 18S AND NON-PARENTS DIRECTED TO THIS MESSAGE:

This survey is only for individuals 18 years and older who care for children under the age of 5 years.

Thank you for your interest. For more on our work, visit us at www.wits.ac.za/coe-human.

DENIED CONSENT PAGE

THOSE WHO DON'T CONSENT DIRECTED TO THIS MESSAGE:

You have been directed to this page because you chose not to participate in the survey.

If this was a mistake, you can follow the link again to complete the survey.

If not, thank you for your time. For more on our work, visit us at www.wits.ac.za/coe-human.
